# J. A. Arrowsmith-Brown

**Published:** 1937

**Authors:** 


### Obituary

### J. A. ARROWSMITH-BROWN, D.S.O.

When this *Journal* was first produced in 1883, under the Editorship of Greig Smith, the firm of J. W. Arrowsmith, of Quay Street, undertook its printing and publication. It will perhaps help one to appreciate how far we have moved since then if it is pointed out that in it were articles disputing Koch's recent discovery of the tubercle bacillus. Thus, for the whole of the fifty-four years during which modern scientific medicine has developed, the firm of Arrowsmith has been to a very considerable extent responsible for the very high standard of production and the regular appearance of our *Journal*. For this we owe a deep debt of gratitude to two men, J. W. Arrowsmith and his nephew J. A. Arrowsmith-Brown. Our obituary notice of J. W. Arrowsmith in 1913 referred to his many-sided character and to the very great share that he had taken in the foundation of Bristol University. His nephew's work, particularly in regard to the University Colston Research Society, has been equally noteworthy. It is with the very deepest sorrow that now, twenty-four years later, we have to record the death of his nephew and successor as head of the firm, James Arnold Arrowsmith-Brown, who died at his home at Abbot's Leigh on 15th June last, at the relatively early age of fifty-four.

His many services to the community have been more suitably related in the public Press, so here we may perhaps try and show what manner of man he was. Whatever he had to do, either in work or play, he did with the utmost keenness, and whether it was the production of a book, the restoration of a church, or the playing of a game of cricket with his family, he always gave of his best with an entire absence of self-advertisement. As is usual with such a character, he was intolerant of hypocrisy and any form of self-seeking. During the War his outstanding success as an officer responsible for the communications of a Division was entirely due to his keenness and unselfishness. Although dealing with a highly technical subject for which he had no particular bent, his tireless energy, good fellowship, and consideration for those under his command rendered his services of the utmost value. As a friend one could argue good-humouredly with him on any subject, and then, not having seen him for a long period, start again where one had left off. Latterly his link with the Medical Profession became a very close one. For seven years, cheerfully and courageously, he carried on in spite of failing health. He tackled the problem of his illness with the same determination that he gave to all else, and refused to give in, rather tending to take on fresh responsibilities when most of us would have felt justified in retiring from active life altogether. This cheerful refusal to accept defeat was also evident during the dark days of the War in his treatment of those who could only see the black side of things.

His life has been held up by the Vice-Chancellor of the University recently as an example to be emulated by the younger generation.

We offer our profoundest sympathy to his widow and family.

